# Order within disorder: Aggrecan chondroitin sulphate-attachment region provides new structural insights into protein sequences classified as disordered

**DOI:** 10.1002/prot.22839

**Published:** 2010-07-30

**Authors:** Thomas A Jowitt, Alan D Murdoch, Clair Baldock, Richard Berry, Joanna M Day, Timothy E Hardingham

**Affiliations:** Wellcome Trust Centre for Cell Matrix Research, University of ManchesterManchester, M13 9PT, United Kingdom

**Keywords:** Aggrecan, chondroitin-sulphate attachment region, SAXS, analytical ultracentrifugation, intrinsically disordered, segmental flexibility

## Abstract

Structural investigation of proteins containing large stretches of sequences without predicted secondary structure is the focus of much increased attention. Here, we have produced an unglycosylated 30 kDa peptide from the chondroitin sulphate (CS)-attachment region of human aggrecan (CS-peptide), which was predicted to be intrinsically disordered and compared its structure with the adjacent aggrecan G3 domain. Biophysical analyses, including analytical ultracentrifugation, light scattering, and circular dichroism showed that the CS-peptide had an elongated and stiffened conformation in contrast to the globular G3 domain. The results suggested that it contained significant secondary structure, which was sensitive to urea, and we propose that the CS-peptide forms an elongated wormlike molecule based on a dynamic range of energetically equivalent secondary structures stabilized by hydrogen bonds. The dimensions of the structure predicted from small-angle X-ray scattering analysis were compatible with EM images of fully glycosylated aggrecan and a partly glycosylated aggrecan CS2-G3 construct. The semiordered structure identified in CS-peptide was not predicted by common structural algorithms and identified a potentially distinct class of semiordered structure within sequences currently identified as disordered. Sequence comparisons suggested some evidence for comparable structures in proteins encoded by other genes (PRG4, MUC5B, and CBP). The function of these semiordered sequences may serve to spatially position attached folded modules and/or to present polypeptides for modification, such as glycosylation, and to provide templates for the multiple pleiotropic interactions proposed for disordered proteins. Proteins 2010. © 2010 Wiley-Liss, Inc.

## INTRODUCTION

Aggrecan is a large glycosylated multidomain protein with both folded and extended sequences readily identified in electron micrographs of single molecules.^1^,^2^ The long-extended sequences contain a high density of attached glycosaminoglycan chains, which are essential to the role of aggrecan as a hydrophilic water-retaining matrix proteoglycan within cartilaginous tissues. These glycosylated domains are present in most other members of the aggrecan family, including versican, neurocan, and brevican. However, their sequences are not highly conserved, and their structure is unknown. Secondary structure analysis of these regions, using common computational algorithms, predicts that they have little organized structure, and disorder prediction programs indicate a high propensity for disorder within these regions (Fig. S1). It has been assumed that these regions are intrinsically unstructured, and their elongation as spacing elements between aggrecan globular domains (evident by EM) has been attributed to the high density of glycosylation, but this has never been studied.

The aggrecan globular domains (G1, G2, and G3) include classic Ig-fold and C-type lectin (LEC) and complement control protein (CCP) sequences and are recognized in rotary-shadowed EM images as globular structures,^3^ and the G1 and G3 domains have specific ligand-binding properties essential for their function in cartilage extracellular matrix.^3^,^4^ The G3 domain has alternatively spliced forms, where the C-type LEC and CCP domains are always present, but two EGF-like motifs are expressed in some spliced forms, which can modify the C-type LEC ligand-binding properties.^4^

In this study, we characterized the structures of two separately expressed parts of the C-terminal regions of human aggrecan; the globular G3 domain (the commonly expressed form lacking EGF-like motifs) and a section of the chondroitin sulphate (CS)-attachment region adjacent to G3. They were chosen as they are based on sequences of similar length, but with quite different predicted structures. There has been extensive recent analysis of disordered protein sequences,^5^–^7^ and the CS-attachment region was also predicted to be disordered (Fig. S1). However, we discovered that the CS-peptide contained an ordered, but flexible structure, which caused it to be significantly stiffened and elongated, and we concluded that this stiffened conformation may be more commonly present amongst other proteins in which sequences identified as disordered have been assumed to have little secondary structure.

## METHODS

### Protein expression and purification

The expression and purification of human G3 domain, containing the LEC, CRP, and tail region, have been described previously.^4^ The human CS-peptide was from the aggrecan CS2 domain (5943–6867 bp of the NCBI reference sequence NM NM_013227.3) and was amplified as cDNA by PCR between primers (5′) CAGTCATATG GAATTCAGTGGCCTACCA, introducing an *Nde* I restriction enzyme site (underlined) and (3′) GCATGG ATCC**TCA**TGTCCCCACATCACTGGT, introducing a stop codon (bold) and a *Bam*H I restriction site (underlined). The PCR product was cloned in the TA vector (Invitrogen), and the sequence was verified and then subcloned into the pET-14b vector (Novagen) at the same sites. The purified plasmid was transfected into BL21 (DE3) cells (Stratagene). IPTG-induced cultures were collected by centrifugation, freeze-thawed, sonicated, and the released 6-his tagged CS-peptide was purified by one-step IMAC using Talon resin (Clontech, Takara Bio Europe) followed by ion-exchange chromatography using a Mini-Q 3.2/3 (GE Healthcare) column and gel filtration on a superdex-200 10/300 column (GE Healthcare). All purification steps were carried out in 10 m*M* Tris–HCl, 150 m*M* NaCl pH 7.4. Elution from the ion exchange was with a linear gradient of NaCl (0–0.5*M*).

For EM studies, a construct (CS2-G3) was generated encoding a 641 amino acid C-terminal section of human aggrecan commencing at amino acid 1893 NP_037359.3, which includes the CS-peptide sequence within a 385 amino acid section of the CS2 region, followed by the G3 domain sequence and a C-terminal 6-histidine tag. The EcoR1-restricted construct had been cloned previously into pBluescript KS (Stratagene).^4^ The CS2-G3 construct was ligated in frame with the α-factor secretion signal sequence of the *Pichia pastoris* vector pPICZαB (Invitrogen) and has a stop signal immediately after the final histidine residue. Zeocin™ resistant colonies were picked by colony hybridization using ^32^P labeled G3 cDNA. The construct was transfected by electroporation into GS115 cells, and the secreted CS2-G3 protein was purified by affinity purification using Talon resin (Clontech) and gel filtration (as mentioned earlier) in 10 m*M* Tris–HCl, 300 m*M* NaCl pH 7.4. All proteins were used for experimental studies immediately following gel filtration.

### Electron microscopy

CS2-G3 was visualized by rotary shadowing TEM. Desalted protein (1 mg/mL) was equilibrated for 24 h in water before being spread on freshly cleaved mica. The samples were snap-frozen in liquid nitrogen and freeze dried in a vacuum (Blazers BAE 120). The samples were then rotary shadowed with evaporated platinum at 6°C and carbon at 90°C. The platinum/carbon replicas were mounted on 400 mesh EM grids and examined with a Philips 301 TEM microscope at 50,000× magnification.

### Analytical ultracentrifugation

Sedimentation of the CS-peptide and G3 domain in 150 m*M* NaCl/10 m*M* Tris–HCl pH 7.4 was performed at 20°C at 50,000 rpm and 40,000 rpm, respectively, in a XL-A ultracentrifuge using an An60Ti 4 four-hole rotor. The boundary was monitored every 90 s at 230 nm. For the urea-unfolding experiments, the CS-peptide was equilibrated overnight in 6*M* urea, 150 m*M* NaCl/10 m*M* Tris–HCl pH 7.4. Sedimentation was performed at 58,000 rpm using an aluminum centerpiece and monitored at 280 nm. Data were interpreted with the model-based distribution of Lamm equation solutions c(s) software Sedfit,^8^ and the data were corrected for standard conditions of water at 20°C using a
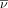
of 0.714 calculated from amino acid composition within Sednterp.^9^ Frictional ratios were calculated directly from the light scattering derived mass and the sedimentation coefficient.

### Homology modeling and solution bead models

To check the validity of the homology model of the G3 domain, solution bead models were generated. The homology model was generated using atomic coordinates for the third CCP motif of CD55, RCSB 1OJW,^10^ and the C-type LEC domain of rat aggrecan RCSB 1TDQ.^11^ The sequences were aligned with human aggrecan LEC and CCP sequences and modeled separately using SWISS MODEL.^12^ The resulting motifs were arranged in several orientations based upon the previous homology model of the domain by Brisset and Perkins^13^ using Pymol.^14^ The solution modeling software SOMO^15^ was used to build multiple bead models of the G3 domain in various conformations. Hydrodynamic parameters generated for the models were compared to the experimental results until a best-fit was achieved.

### Multiangle light scattering

CS-peptide and G3 domain were chromatographed on a Superdex-200 24/300 gel filtration column (Amersham Pharmacia Biotech) in 150 m*M* NaCl/10 m*M* Tris–HCl pH 7.4, driven by a Dionex BioLC HPLC at 0.71 mL/min, and passed through a Wyatt EOS 18-angle laser photometer with the 13th detector replaced with a Wyatt QELS detector (for the measurement of hydrodynamic radius) and also through a Wyatt Optilab rEX refractive index detector. The hydrodynamic radius, molecular weight moments, and concentration of the resulting peaks were analyzed using Astra 5.2.

### Circular dichroism

CS-peptide was purified (as above) by gel filtration chromatography in 150 m*M* NaCl/10 m*M* Tris–HCl pH 7.4 before loading at 5 and 10 μ*M* into a 0.5-mm path-length cuvette. Spectra were monitored on a Jasco J810 spectrometer between 260 and 190 nm with a 0.2-nm step and 10 averages. Urea titration, experiments were performed by dissolving urea in 200 μL of 10 μ*M* peptide to achieve final concentrations of 2, 4, 6, and 8*M* urea, and, after 60-min equilibration, spectra were taken between 255 and 210 nm at 20°C. All spectra were corrected from instrument units to molar units.

### Small angle X-ray scattering analysis of CS-peptide

Small angle X-ray scattering (SAXS) data for CS-peptide (3.2 mg/mL) were collected on EMBL beamline X33 at the light-source facilities DORISIII at HASYLAB/DESY.^16^ Data were collected on a MAR345 image plate detector using a 60-s exposure time and 2.4-m sample-to-detector distance to cover a momentum transfer interval 0.10 nm^−1^ < *q* < 5.0 nm^−1^. The modulus of the momentum transfer is defined as *q* = 4π sin θ/λ, where 2θ is the scattering angle and λ is the wavelength. The *q* range was calibrated using silver behenate powder based on diffraction spacings of 58.38 Å. The scattering images obtained were spherically averaged using in-house software, and buffer scattering intensities were subtracted using PRIMUS. Molecular mass estimates were obtained by normalizing scattering to BSA. The *R*_g_, forward scattering intensity, and 1D intraparticle distance distribution function *p*(*r*) in real space were evaluated with the indirect Fourier transform program GNOM.^17^

## RESULTS

### Characteristics of human aggrecan CS-peptide and G3 domain

**Figure 1:**
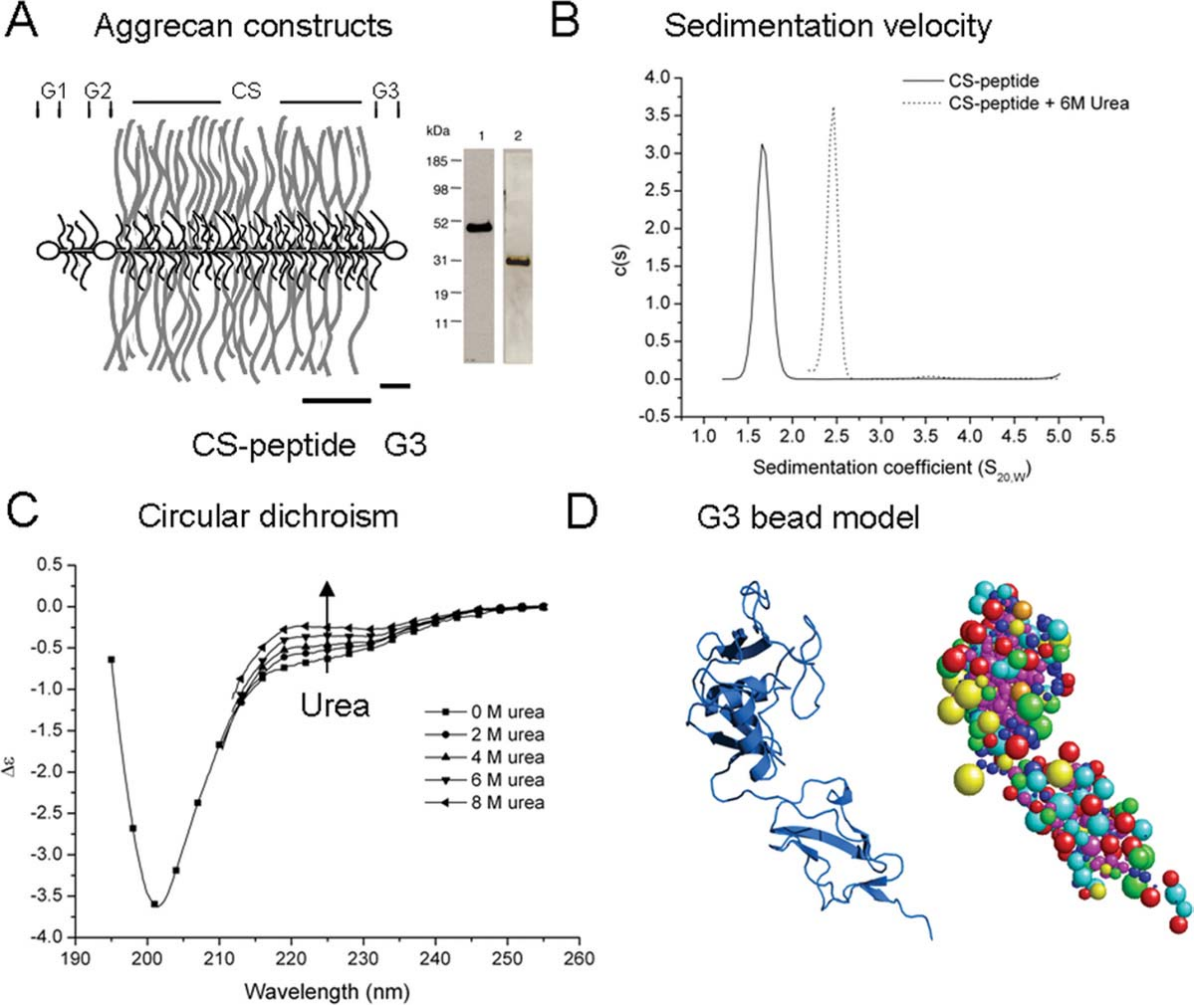
**A:** Constructs from human aggrecan C-terminal were made and designated G3 (for the globular G3 domain encoding the LEC/CRP motifs) and CS-peptide (encoding a 31 kDa segment of the CS-2 glycosylation region). The constructs are roughly equivalent in molecular size, but the CS-peptide (lane 1) has an anomalous migration on SDS-PAGE compared to G3 (lane 2). **B:** Sedimentation velocity of the CS-peptide ±6 M urea. The *X*-axis represents sedimentation coefficient distributions corrected for standard conditions. **C:** Circular dichroism spectra of the CS-peptide. A urea titration causes an increase in the contribution of the PPII spectra at 222 nm. **D:** A homology model of the G3 domain was constructed using rat aggrecan LEC domain and the CRP motif from CD55. The resulting model generated through SWISS-MODEL is represented here as a bead model using the suite of programs SOMO. The resulting hydrodynamic predictions compare well with the experimental results giving confidence in the structure.

The globular G3 domain (25,700 Da, 256 amino acids) was expressed in mammalian HEK 293 cells and the CS-peptide (30,827 Da, 308 amino acids), which formed part of the highly glycosylated region of human aggrecan, was expressed in a nonglycosylated form in *E. coli* [Fig. 1(A)]. To determine the overall hydrodynamic characteristics of the CS-peptide and G3 domain in physiological buffers, the mass of each purified recombinant protein was determined using size-exclusion chromatography combined with multiangle laser light scattering (MALLS/SEC) (see Fig. S2). The results showed that both proteins were monomeric in solution with weight-averaged molecular weights comparable to the predicted sequences (26,800 ± 700 Da for G3 and 28,670 ± 1787 Da for CS-peptide). However, the elution position of the CS-peptide was much earlier than the G3 and, thus, despite the similar molecular weights, the hydrodynamic radius of the CS-peptide was much larger than that of the G3 domain (see Fig. S2).

Further analysis of their behavior in solution showed the CS-peptide to have a very low-sedimentation coefficient of 1.67 ± 0.11 S compared to 2.38 ± 0.14 S for the G3 domain and a high-frictional ratio (*f*/*f*_0_), 2.0 compared to 1.3 for the more compact and globular G3 domain (see Table I). The CS-peptide therefore exhibited none of the properties of a compact globular domain, like G3, and the high (*f/f*_0_) showed that it was also not a random coil. The structure most comparable with the hydrodynamic data (Table I) from the sedimentation and the MALLS/SEC analyses suggested that the CS-peptide was highly asymmetric and elongated in solution. The G3 domain in these analyses showed behavior of a classic globular protein as predicted from its sequence homology with other folded proteins. These initial observations on the CS-peptide were unusual in finding such strong evidence of structure within the unglycosylated CS-peptide under nondenaturing conditions.

**Table I:** Hydrodynamic Data Collected for the CS-Peptide and G3 Domain

	Mass^a^ (Da)	Mass^b^ (Da)	Sed. Coeff. (S_20,W_)^c^	*f/f*_0_^c^	*R*_h_ (nm)^c^	*R*_g_^d^ (nm)	Max dimension^d^ (nm)
CS-peptide	30,827	28,670 ± 1,787	1.67 ± 0.11	2.01	3.99 ± 0.28	4.71	15.8
G3	25,700	26,800 ± 700	2.38 ± 0.14	1.31	2.57 ± 0.17	N/D	N/D

The molecular weights were from (a) sequence and (b) light scattering whilst the values for sedimentation coefficient, *R*h and frictional ratio were obtained from (c) sedimentation velocity. *R*g and particle dimensions (d) using SAXS were only obtained for the CS-peptide.

### Urea denatures the CS-peptide structure

The very low-sedimentation coefficient for the CS-peptide (1.67 S) suggested that it was elongated and potentially rodlike. To understand if these properties were based on some organized secondary structure stabilized by hydrogen bonds, the sedimentation coefficient was redetermined under denaturing conditions in 6*M* urea. This resulted in an increase in sedimentation from 1.67 to 2.40 S [Fig. 1(B)], which suggested a collapse of the elongated structure to become more comparable to a random coil (calculated to be 2.1 S for a 30 kDa polypeptide^18^). This large effect of urea thus suggested that it caused the disorganization and loss of secondary structure and provided strong evidence that the CS-peptide had a semiordered structure in the native state.

### The CS-peptide structure is compatible with an ensemble of β-turn and polyproline helix type-II conformations

Further evidence of ordered secondary structure in the nonglycosylated CS-peptide was obtained by circular dichroism spectroscopy (CD), which showed distinct features, including a strong minima at 204 nm and a shoulder at ∼223 nm [Fig. 1(C)] with some of the characteristics reported for a repeating β-turn^19^ and left-handed polyproline helix type II.^20^,^21^ The reasonably high-proline content of the CS-peptide (8%) (Fig. S3) could lend itself to adopting units of left-handed polyproline type-II helices (PPII). These secondary structure motifs have become increasingly identified within sequences previously interpreted as intrinsically unfolded, especially those with a high-proline content; although they are also found within globular domains^22^,^23^ and short polypeptides,^24^ they have been proposed to be a common structural feature of unfolded proteins.^21^,^25^,^26^ Although the CD signal for CS-peptide contained the minima (204 nm) characteristic of PPII helices, it lacked the more prominent positive shoulder associated with pure left-handed PPII conformations. Titration of the peptide with urea resulted in a characteristic apparent increase in the contribution of the PPII ellipticity maxima at 220 nm, which is likely to result from a decrease in the contribution to the CD signal of other structures such as β-turns and an increase in the contribution of the PPII helices.^27^

Attempts to assess if structure in the CS-peptide might be detected by the more recently developed structural algorithms were unsuccessful, as the neural network and multiple alignment algorithm BETATPRED2^28^ predicted 96% β-turn probability and contrasted with PSIPRED^29^ secondary structure similarity search engine, which suggested 98% coil and 2% β-structure. These analyses did not therefore add to the interpretation of the CD spectrum, which showed clear evidence of some order that might be related to left-handed PPII helices and repeating β-turns in the native CS-peptide structure

The CD analyses together with the sedimentation behavior suggested that left-handed PPII helices and repeating β-turns are the predominant secondary structures within the peptide. The loss of stiffness and change in CD spectrum with the addition of urea is due most likely to a loss of β-structures and backbone hydrogen bonds, yielding a more flexible conformation. The absence of the sedimentation characteristics of a true random-coil in 8*M* urea and the accompanying residual CD signal suggested that this more flexible CS-peptide conformation retained short segments of PPII helices.

### G3 domain homology model

To validate the experimental approach used with the CS-peptide, the same principles were used to derive a structural model for the aggrecan G3 domain. This, in sharp contrast to the CS-peptide, behaved as a classic globular protein, and the results from the biophysical analyses were combined to generate a homology-based hydrodynamic bead model. This was compared to a homology model previously proposed by Brissett and Perkins.^13^ We incorporated more recently available rat aggrecan C-type LEC and human complement regulator CD55 as a template, and a homology model generated using SWISS MODEL was converted to a bead model using the software SOMO [Fig. 1(D)]. This predicted a hydrodynamic radius of 2.42 nm for the homology model, which is close to that obtained experimentally and showed that the G3 domain model is highly compatible with its hydrodynamic properties. These clear results for G3 gave confidence in the analysis of the CS-peptide, which showed its biophysical properties to predict a stiffened and elongated structure.

### Small-angle X-ray scattering of CS-peptide confirms an elongated structure with flexible segments

**Figure 2:**
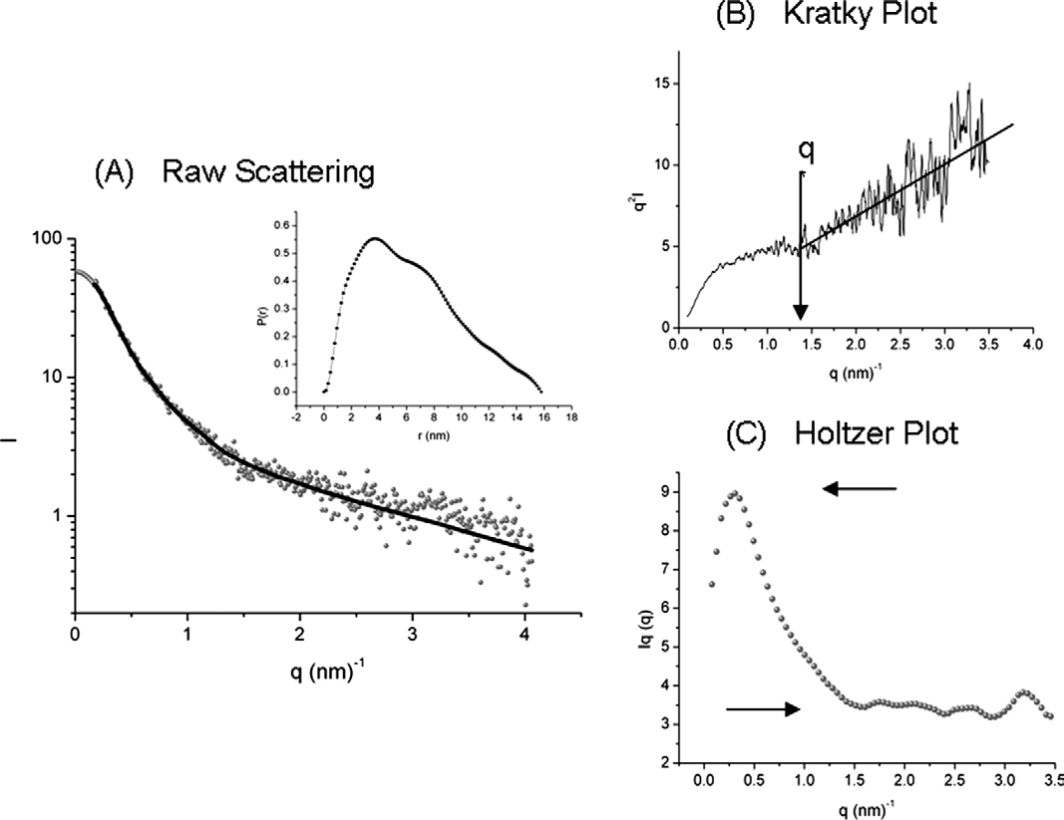
Small angle X-ray scattering (SAXS) analysis of the CS-peptide: (**A**) plot of log intensity of the scattering of CS-peptide against the scattering vector (*q*) and the inset shows the pair distribution function [*p*(*r*)]. The *D*_max_ of the CS-peptide of 15 nm can be estimated when *p*(*r*) reaches zero. **B:** The data represented as a Kratky plot, variation of *q*^2^*I* with respect to *q*. The plot shows a next-neighbor average of raw scattering data, which produces a plot with an intersection of the Kratky plateau and linear high-*q* termed here as q*, which corresponds to Kuhn segment length. A Kratky plot with linearly increasing values at high-*q* is representative of a segmentally stiffened polypeptide. **C:** A Holtzer plot was used to determine peptide mass per unit length due to the ambiguous positioning of the Kratky plateau. The arrows represent the maximum curve height and the asymptotic plateau. Assuming a monodisperse sample, the ratio of these values is related to the segment length.

Having generated evidence for the CS-peptide having a native structure in solution that was highly asymmetric and elongated, we explored this further by SAXS. From the raw scattering with respect to *q* (the scattering vector) [Fig. 2(A)], the radius of gyration (*R*_g_) of the CS-peptide was obtained from its angular dependence using a Guinier approximation [log *I* vs. *q*^2^, where *I* is the scattering intensity and *q* = (4π/λ sin θ)], which, at low *q*, is independent of conformation and gives an indication of the shape. The results gave the CS-peptide a large *R*_g_ of 4.71 nm and a ratio *R*_g_/*R*_h_ of 1.18, which indicated it to be far from globular (globular *R*_g_/*R*_h_ ∼ 0.78). However, it was also far from rigid rod behavior (*R*_g_/*R*_h_ ∼ 2), but was compatible with the molecule being elongated and segmentally flexible.

Additional information on the conformation was gained from the analysis of the intensity of scattering away from the centre of mass. Kratky and Porod^30^ established that molecules with a polypeptide chain, resembling a wormlike chain pattern, or a more rigid rod, showed distinct features in a plot of *q*^2^*I* against *q* (Kratky plot) [Fig. 2(B)]. The CS-peptide had a typical signature for a segmentally flexible, or rodlike chain, with a plateau at intermediate *q*, and an increasing linear relationship at higher *q*.^30^,^31^ This distinguished it from (a) globular proteins, which have a characteristic hump at intermediate *q* decreasing to the origin at higher *q* values, and (b) flexible molecules, which tend to have no intermediate feature and a signal declining to background.^32^,^33^ The detail of the SAXS scattering thus also confirmed that the CS-peptide had some order in its structure.

From the analysis of the intensity of scattering over a broad range of *q*, the model for the CS-peptide structure most compatible with the data was an overall elongated, but flexible structure formed from a wormlike chain of stiffened segments. From the data, the intersection between the linear scattering at high *q*, and the plateau, termed q* [Fig. 2(B)], was used to estimate the length of the stiffened segments of polypeptide chain within the wormlike chain model. *R*_g_ is related to the number of homogeneous linear polymers (*N*), with a segment length (*l*_s_) by Eq. (1),^31^ which is valid for monodisperse polymers in which q* < 2 Å, q* = 12/π*l*_s_.^34^


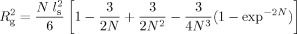
(1)

The wormlike chain can be represented as a sequence of *N* repeating rigid rods with flexible linkers. The rods have a length, *l*_s_, and therefore the contour length of the molecule *L* is *L* = *Nl*_s_. From Figure 2(A), the transition q* is 1.48 Å, which corresponds to a segment length of 2.58 nm and a predicted *N* of 20. This suggested a relatively large number of segments; however, some overestimation of the number of segments may arise from uncertainty in the determination of the Kratky plateau. A further estimate of the segment length was obtained from a Holtzer plot for the CS-peptide [Fig. 2(C)] in which a peak at shorter *q* and a decrease to an asymptotic plateau at higher *q* is characteristic of a stiffened coil. The relationship between *R*_g_ and peak height shows that the sample is monodisperse.^31^ This then allows us to gain an estimate of the number of chain segments from the ratio between peak height and plateau.^35^,^36^ Using the calculated ratio (*p*_r_) of 2.53 and the plot of *p*_r_ versus *N*,^35^ the results suggested that CS-peptide could be represented as a semiflexible chain of 12 segments of 3.3 nm each. This would equate to a molecule with an overall contour length of 39.6 nm. Modeling the CS-peptide as a wormlike chain of stiffened segments thus gave estimates of segment length between 15 and 25 amino acids long.

As a comparison, an extended 308 amino acid polypeptide based on an amino acid contour length of 0.4 nm and with no defined secondary structure would have a contour length of 123.2 nm.^37^ This contrasts with the CS-peptide's estimated contour length in solution of 39.6 nm, which is only one third of the fully extended polypeptide length and corresponds to a mass per unit length of 770 Da nm^−1^ compared to 250 Da nm^−1^ for a fully extended polypeptide chain of the same amino acid composition. Taken together, the SAXS analysis thus suggested a segmentally flexible polypeptide backbone based on significant secondary structure and forming an elongated conformation with a high-mass per unit length. It thus provided independent evidence to corroborate and extend the velocity sedimentation and CD results.

### Comparison of the CS-peptide with native aggrecan

To understand how the predicted structure of the unglycosylated CS-peptide corresponded to the structure of the peptide when fully glycosylated, we compared its dimensions with single-molecule AFM images and rotary shadowing electron micrographs of bovine aggrecan.^1^,^2^ These studies show a average mature contour length of ∼272 nm for the whole CS-attachment region containing 1501 amino acids and with ∼100 CS chains attached, which equates to an average mass/length of 585 Da nm^−1^. This is lower than the 770 Da nm^−1^ calculated for the segmental CS-peptide structure and may suggest that the fully glycosylated CS region, in addition to being less flexible, is rather more extended than when unglycosylated, although it is still only 45% the length of a fully extended sequence.

**Figure 3:**
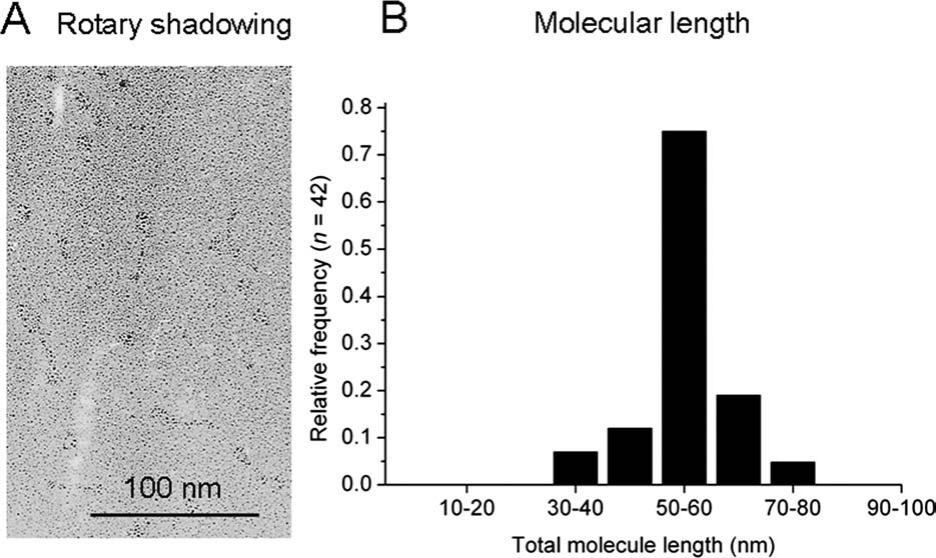
**A:** Rotary shadowing electron microscopy images of CS2-G3. The images appear as a globular domain (G3) with an attached extended tail (CS2). The bar represents 100 nm. **B:** The total molecular length distribution indicates a mean molecular length of 57 ± 5.2 nm for the globular domain and the tail and is suggests a contour length of 48 ± 4.4 nm for CS2 (the tail) with a mass per length of 802 Da nm^−1^.

To gain a further independent estimate of contour length for the CS-peptide and the awareness that various preparative artefacts may cause some uncertainty in assigning accurate dimensions to images of highly glycosylated aggrecan molecules, we investigated the peptide by EM. However, the CS-peptide on its own did not yield definable images. We therefore expressed a construct (CS2-G3) (Fig. S4) containing the G3 domain and the naturally adjacent 385 amino acids of the CS attachment region. The protein product was expressed in yeast and was glycosylated, but lacked the CS chains. The extent and type of glycosylation was examined by cleaving N-linked sugars with PNGaseF/EndoF, expression of a cell free construct and staining with periodic acid Schiff staining (Fig. S4). The results indicate that there was ∼25–30 kDa of O-linked sugars, and the one possible N-linked site was glycosylated. The presence of the globular G3 domain and glycosylation of the CS region enabled images to be observed [Fig. 3(A)] using rotary shadowing EM in which it appeared as a globular domain with an extended side arm. The overall dimension was 57.0 ± 5.2 nm [Fig. 3(B)], with the side arm contour length ∼48 ± 4.4 nm. Interpreting the side arm as representing the CS2 sequence and allowing for the fact that it contained the CS-peptide (30 kDa) and 8 kDa of additional CS2 sequence, it was proportionately longer and comparable to the predicted length of the CS-peptide (39.6 nm). For the CS2-G3 images, the length of the CS2 segment equated to a mass/length of 802 ± 69 Da nm^−1^, which is comparable to the unglycosylated CS-peptide (770 Da nm^−1^) but both were less extended than the fully glycosylated aggrecan 585 Da nm^−1^). This may imply that the CS2-G3 construct, expressed in yeast cells (*P. pastoris*), was elongated similar to the CS-peptide expressed in *E*. *coli*, but that attachment of the many long CS chains (averaging 1 every 15 amino acids) to aggrecan in mammalian cells extends the conformation further. The EM appearance of the CS2-G3 protein clearly showed a globular G3 domain and an elongated CS-peptide structure of similar dimensions to the CS-peptide structure we derived from the biophysical analysis.

### The CS-peptide shares structural similarities with other elongated and linker sequences in other proteins

A major difficulty in identifying potentially elongated sequences sometimes referred to as linker sequences, amongst those lacking classically folded motifs, is the fact that the sequences generating them are not highly conserved.^38^ The CS-peptide in aggrecan is less conserved amongst different mammalian species (61–73% conserved) than the adjacent-folded G3 domain (92–94% conserved). The structure we identify in CS-peptide is thus suggested to tolerate many amino acid changes and still retain its stiffened and elongated conformation. The key feature of such sequences may thus be the absence of motifs that drive the formation of folded structures, rather than the presence of specific sequences that generate elongated stiffened structures.

**Figure 4:**
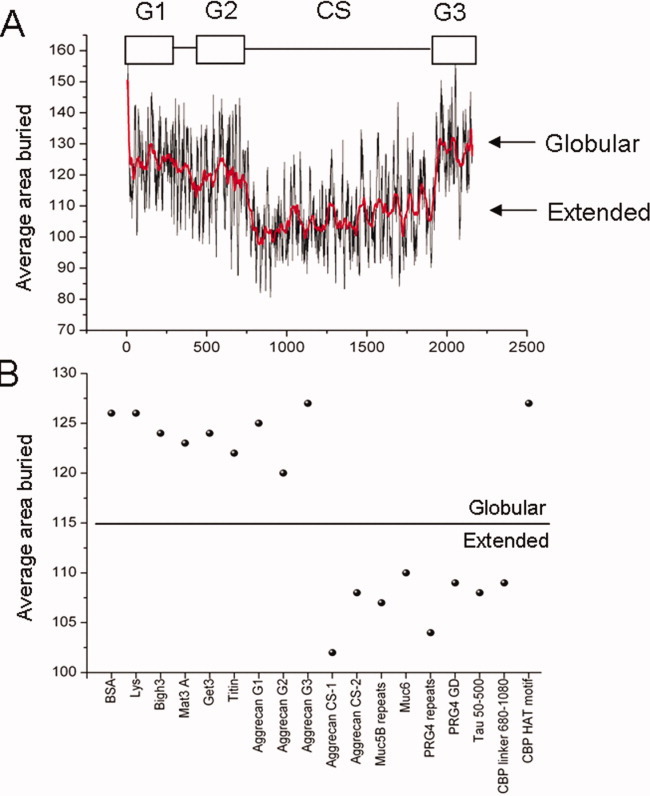
A: Bioinformatics analysis of the full sequence of aggrecan based on the average area buried upon folding for each amino acid. This simple algorithm developed by Rose *et al.*^40^ is able to highlight extended sequences. The domain architecture within aggrecan can clearly be distinguished as an overall decrease in the average area buried within the extended sequences corresponding to the chondroitin sulphate attachment (CS)-region. The red line is an 80-point Savkitzky–Golay average. By applying the same algorithm to other proteins and protein domains, there is a clear distinction in the score obtained between extended and globular characteristics (**B**). BSA, lysozyme (Lys), Bigh3, matrilin-3 A domain (Mat3 A), Get 3, titan, and the globular domains of aggrecan and the HAT motif of CBP, all produce an average domain score of >115, whilst the extended regions of aggrecan, muc5B tandem repeats, muc6, PRG4 tandem repeats and glycosaminoglycan attachment region (PRG4 GD), and the linker region of CBP, all produce scores <115. This analysis identifies a structural class distinction for some glycosylated and linker sequences and may correlate with stiffened and extended conformations.

To determine whether the CS-region of human aggrecan has common structural features present in other multidomain proteins, several proteins were analyzed using the structural prediction programs within the suite of programs in Protscale.^39^ Using the highly glycosylated sequences of aggrecan, MUC5B mucin, and PRG4 and the nonglycosylated linker sequences of CBP (CREB-binding protein), we were able to show differences between extended and globular domains. The algorithms that showed the greatest differences between globular domains and extended regions were (a) the average area buried,^40^ which estimates the area buried upon refolding of the molecule based on hydrophobicity (Fig. 4 and S5); (b) the normalized β-turn content,^41^ which is an empirical relationship between the length of the side chain and the preference for certain structures; and (c) the percentage of accessible residues,^42^ which assesses the statistical accessibility of the residues to solvent, based on a subset of crystal structures.

The bioinformatic analysis highlighted the boundaries between the regions known to be globular and those that are glycosylated and/or extended. These regions have a high frequency of hydrophilic residues, which makes opportunities for H-bonding between adjacent amino acid side chains more common and thereby may increase overall chain stiffness. These areas are also statistically more inclined to be accessible to solvent. The β-turn prediction by Levitt^41^ showed that these areas were more disposed to β-turn conformation, supporting the neural net algorithm analysis. The difference between these regions and classically folded domains is clearly identified in comparison with well-characterized globular proteins [Fig. 4(B)] and could form a class distinction between recognized globular regions and extended linker/glycosylation sequences. Interestingly, disorder prediction identified the CS-attachment region of aggrecan as being disordered (Fig. S1), but could not clearly distinguish the extended linker domains in CBP.

## DISCUSSION

The CS attachment region of aggrecan has historically been assumed to be intrinsically unstructured, and, furthermore, in amino acid composition and lack of hydrophobic clusters, it has much that is now recognized to be in common with other intrinsically disordered protein sequences.^38^ Also, in common with other disordered sequences, it is less well conserved than the corresponding globular domains amongst vertebrate aggrecans (as noted earlier). The analysis of the recombinant expressed CS-peptide by a range of biophysical techniques, including molecular weight, *R*_g_ and *R*_h_ determinations, SAXS analysis, CD spectrum, sedimentation coefficient, and its sensitivity to 6M urea, showed that it formed a stiffened and elongated structure in solution in the absence of glycosylation. The extent of this semiordered structure detected within the native CS-peptide was surprising, but could be represented by a segmentally flexible chain or wormlike model, which also matched EM dimensions of expressed sequences (CS2-G3) and was compatible with images of fully glycosylated aggrecan molecules. From these images, it appeared that glycosylation resulted in some increased extension (∼25%) of an intrinsically stiffened, elongated structure.

**Figure 5:**
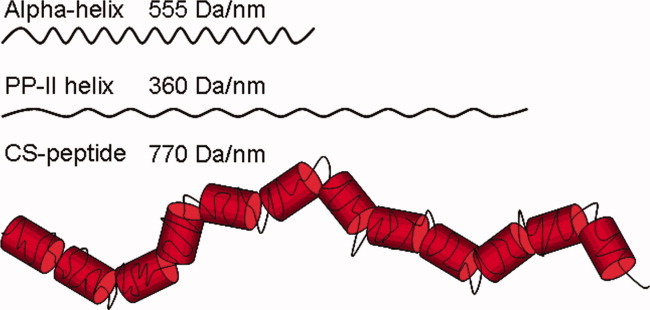
Schematic comparison of the model predicted for CS-peptide with polypeptides in α-helical and PPII helical conformations. The CS-peptide in solution is predicted to be a semiflexible molecule, significantly stiffened, and elongated, compared to a polypeptide in a fully disordered or random coil orientation and with much higher mass/length than pure α-helical or PPII helical structures. It is suggested to contain abundant PPII-like and β-turn elements with local sequences of higher mass/length caused by hydrogen bonds creating segments with restricted flexibility interspersed by more flexible peptides. This creates the high-average mass per unit length of 770 Da nm^−1^ predicted for the CS-peptide, which contrasts with pure PPII or α-helical polypeptides and indicates the increased structural organization present within the CS-peptide. [Color figure can be viewed in the online issue, which is available at wileyonlinelibrary.com.]

Although our analysis suggests that the structure may contain β-turns and PPII helices, it is important to note that the level of structure we detected within the CS-peptide far exceeded that of a PPII-like polypeptide chain alone, which only has a mass/length of ∼360 Da nm^−1^ (established using crystal structure 2HO2^43^ (see Fig. 5). The predicted mass per unit length of the CS-peptide from biophysical analysis and that calculated from EM clearly shows that it contains more secondary structure than is associated with disordered sequences and which is not described by current classifications. However, it is recognized that disordered proteins may contain varying levels of suborder,^5^ which has been interpreted as arising from peptide backbone and side chains participating in many weak interactions stabilized by hydrogen bonds between groups and with water,^44^ but the full structural consequences of these have not been described. These interactions together may generate many closely related semiordered structural conformations that are equally energetically favorable, such that the structure flips between them on a rapid time scale. This concept is compatible with the stiffened CS-peptide structure and its sensitivity to urea. An interesting parallel is found in the polysaccharide hyaluronan, which is much more extended and stiffened than predicted by known rotation about its glycosidic bonds, and there is experimental evidence that it rapidly explores a range of conformers stabilized by hydrogen bonds and which are sensitive to urea.^45^ Comparable structural analogies have therefore been observed in other biological polymers.

Based on our detailed biophysical analysis, the CS-peptide presents a more ordered example of a disordered sequence, and this form of elongated structure may be more common than is currently recognized in other “linker” sequences in proteins. It is estimated that 50% of known protein sequences lack recognized conserved motifs that favor stable folding^6^ and are not currently predicted to have any ordered structure. Therefore, it may be that various forms of this semiordered stiffened and elongated conformation found in CS-peptide are present in other expressed protein sequences.

The semiordered structure we detected in the CS-peptide expressed in a nonglycosylated form raises a number of questions regarding its likely function. In secreted proteins, the presence of these structures may have consequences on the intracellular events of processing, from translation in the RER, through translocation and glycosylation in the Golgi and secretion from the cell. In common with observations on other disordered protein domains, the recombinant CS-peptide was poorly expressed and secreted in mammalian (COS) cells, but the efficiency was greatly enhanced when it was expressed linked to any of the folded elements of the G3 domain.^4^ This suggests that the semiordered CS-peptide on its own lacks the interaction with chaperones necessary for nascent protein translocation and may initiate an unfolded protein response. However, the secondary organization of CS-peptide and its lack of interaction with chaperones may be important in facilitating the rapid glycosylation of aggrecan within the Golgi.^3^

The concepts evolving in the function of disordered protein sequences has placed great emphasis on the ability of flexible dynamic structures to facilitate the pleotropic interactions of one sequence with several target ligands,^38^ and there are many examples of this amongst intracellular regulatory proteins. The distinct features we identify here in the aggrecan CS-peptide in the generation of a flexible, dynamic, but elongated structure, may provide a new function. For as a link between folded domains, it would hold them at a fixed distance and thus determine their spatial position, which may be of key importance in matching intermolecular interactions with topography.^46^ It could also function in this way in cell-surface receptors to present extracellular domains on extended stalks away from the membrane surface for ligand interaction, or intracellularly, to present domains for cytoplasmic signaling.

Here, we have shown that the heavily glycosylated domain of aggrecan has evolved to have amino acid sequences, which in solution form a stiffened, elongated conformation. This form of semiordered polypeptide structure is undetected by common structural prediction methods and it may be commonly found amongst the vast range of “disordered” protein sequences in the genome. Based on the current lack of bioinformatic tools to enable their detection, evidence for this will depend on further biophysical analysis of expressed sequences.
