# Supplementary material for: Order within disorder: Aggrecan chondroitin sulphate-attachment region provides new structural insights into protein sequences classified as disordered
Source: Proteins. 2010 Jul 30;78(16):3317–27. doi: 10.1002/prot.22839 (PMC3546398; doi:10.1002/prot.22839)
Supplement: Supplementary file 1 [file prot0078-3317-SD1.pdf]

Supplemental 1: Disorder prediction

A. Domain organisation of aggrecan

G1 domain residues	1-327
IGD residues	328-453
G2 domain residues	454-652
KS region residues	653-732
CS region residues	733-1911
CS-1	(733~1275)
CS-2	(1276~1911)
G3 domain residues	1912-2162

B. Disorder prediction of aggrecan using RONN

Further information on the algorithms can be found in these references:

Yang,Z.R., Thomson,R., McMeil,P. and Esnouf,R.M. RONN: the bio-basis function neural network technique applied to the dectection of natively disordered regions in proteins Bioinformatics (2005) 21:3369-3376
Thomson,R. and Esnouf,R.M. Prediction of natively disordered regions in proteins using a bio-basis function neural network. Lecture Notes in Computer Science, (2004) 3177/2004, 108-116
Thomson,R., Hodgman,C.T., Yang,Z.R. and Austin,K.D. Characterising proteolytic cleavage site activity using bio-basis function neural networks. Bioinformatics, (2003) 19, 1741-1747

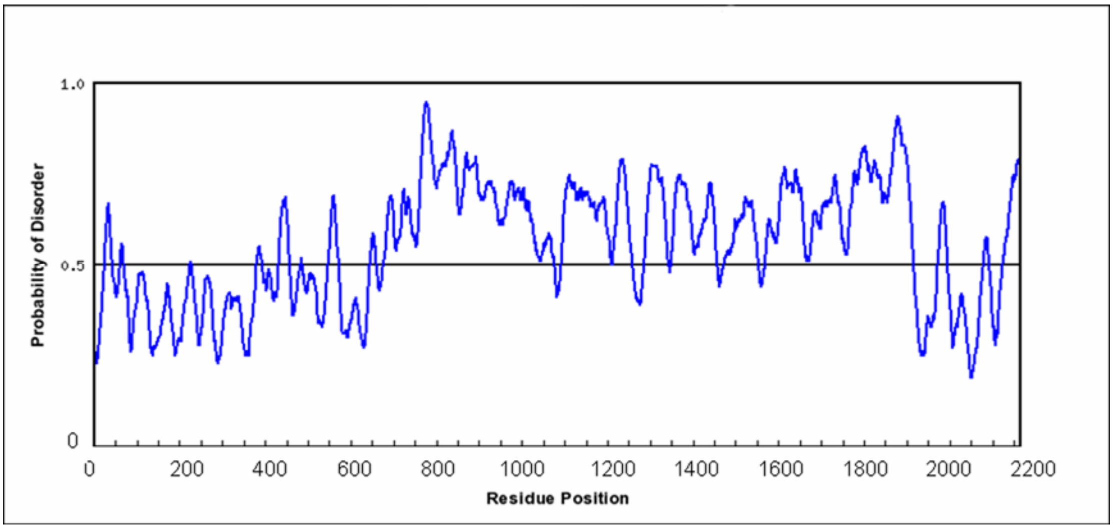

C. Disorder prediction of aggrecan using DiSEMBL 1.5

The score has been averaged with a 20 amino acid average (red-line).

Further information on the algorithms can be found in these references:

Linding, R. Jensen, L.J. Diella, F. Bork, P Gibson T.J. and Russell R.B. Protein disorder prediction: implications for structural proteomics Structure (2003) Vol 11, Issue 11,

Coils

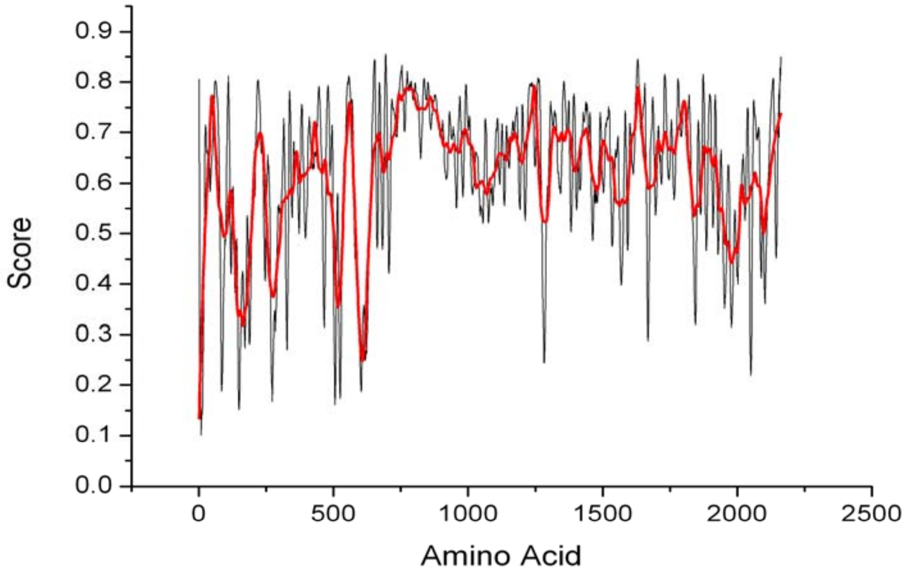

Rem465

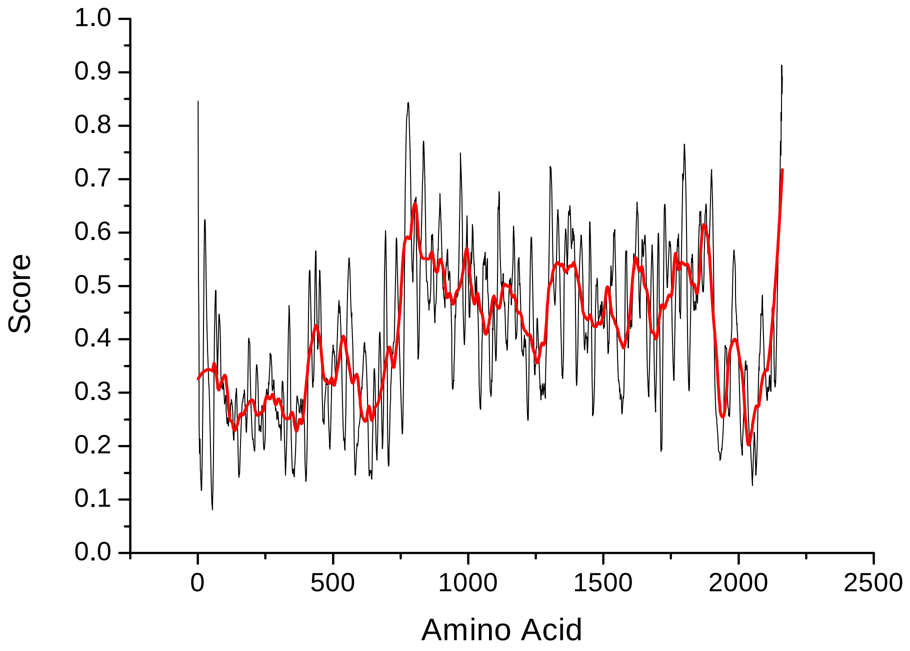

D. Disorder prediction of aggrecan using Foldindex

Further information on the algorithms can be found in these references:

Prilusky, J., Felder, C.E., Zeev-Ben-Mordehai, T., Rydberg, E., Man, O.,. Beckmann, J.S., Silman, I., and. Sussman J.L, FoldIndex©: a simple tool to predict whether a given protein sequence is intrinsically unfolded. (2005), Bioinformatics.

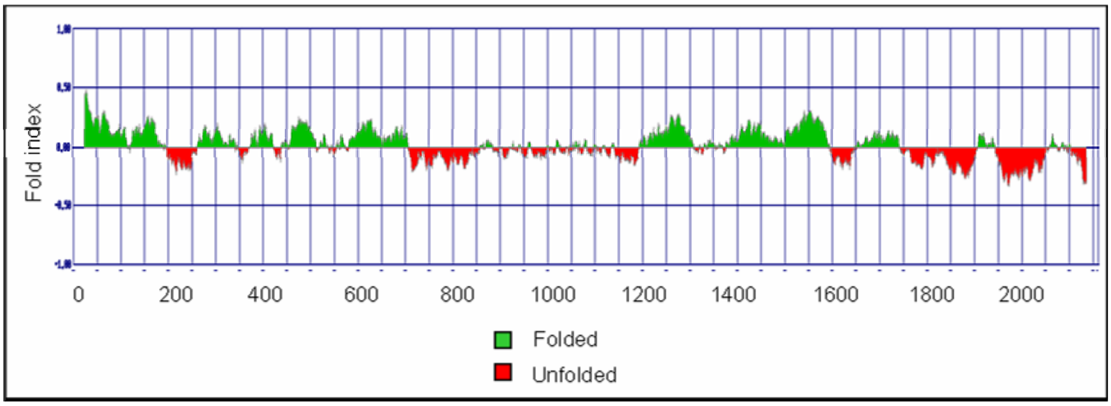

E. Disorder prediction of the CS-peptide using Disopred

Further information on the algorithms can be found in these references:

Ward JJ, Sodhi JS, McGuffin LJ, Buxton BF and Jones DT Prediction and functional analysis of native disorder in proteins from the three kingdoms of life J. Mol. Biol, (2004) 337, 635-645.

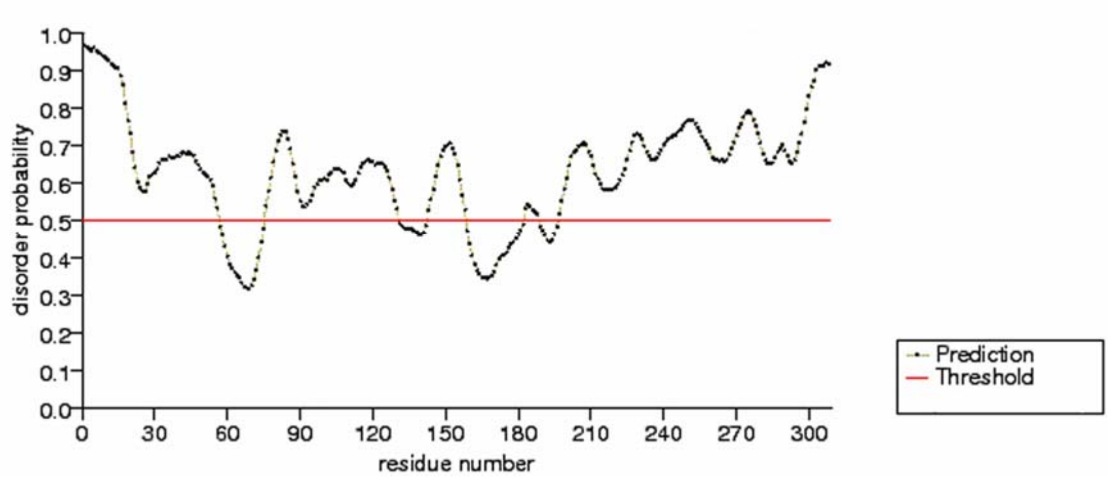

F. Disorder prediction of the CS-peptide using PONDR

Further information on the algorithms can be found in these references:

Prediction of boundaries between intrinsically ordered and disordered protein regions.
Radivojac P, Obradovic Z, Brown CJ, Dunker AK., Pac. Symp. Biocomput. 2003, 8, 216-227.
Exploiting heterogeneous sequence properties improves prediction of protein disorder.
Obradovic Z, Peng K, Vucetic S, Radivojac P, Dunker AK., Proteins. 2005,61 Suppl 7,176-82.
Predicting protein disorder for N-, C-, and internal regions.
Li, X., P. Romero, M. Rani, A. K. Dunker, and Z. Obradovic, Genome Informatics, 1999, 10:30-40.
Sequence complexity of disordered protein.
Romero, P., Z. Obradovic, X. Li, E. Garner, C. Brown, and A. K. Dunker, Proteins: Struct. Funct. Gen., 2001, 42:38-48.
Sequence data analysis for long disordered regions prediction in the calcineurin family.
Romero, P., Z. Obradovic, and A. K. Dunker, Genome Informatics, 1997, 8:110-124.

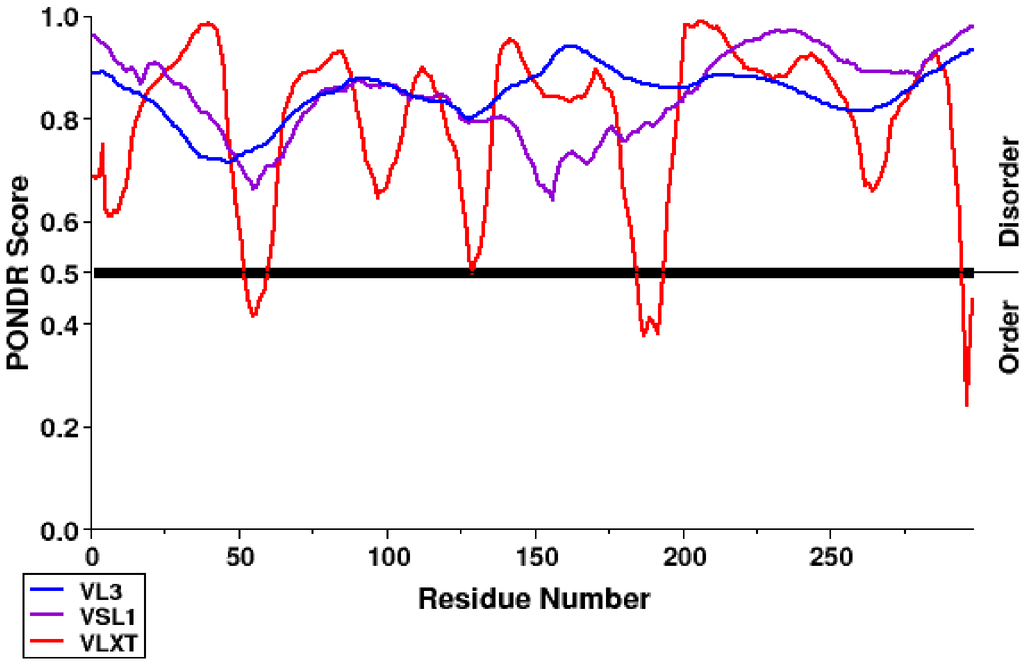

S1: Disorder prediction programs using the whole sequence of aggrecan (A, B, C and D), and the CS-peptide sequence alone (E and F). The amino acid domain organisation of aggrecan is listed in section A. Using RONN (B) and DiSEMBL (C) (Rem465 algorithm), the domain organisation of aggrecan can be clearly seen, with the CS-region predicted to be disordered. This is not so clearly demarked by using Foldindex (D), which predicts a larger extent of the CS region to be folded. Both Disopred (E) and the VL3, VSL1 and VLXT disorder algorithms within PONDR (F) are used on the CS-peptide sequence (see supplemental S3) and indicate that the CS-peptide is largely disordered.

Supplemental 2

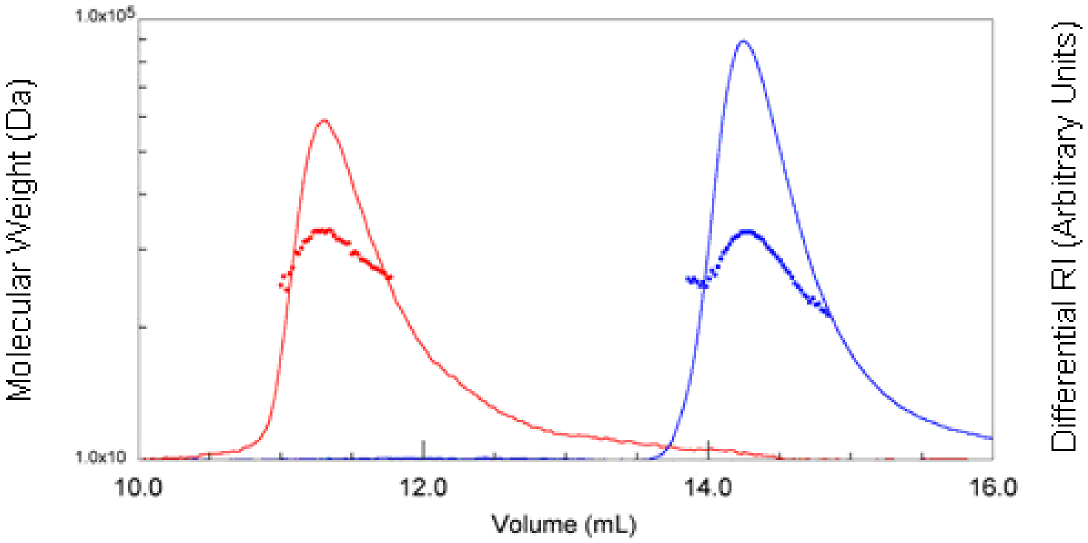

S2: Multi-angle laser light scattering of the both CS-peptide (red) and aggrecan G3 (blue). Proteins were eluted from a Superdex 200 10-300 gel filtration column (GE healthcare) before passing through both refractometer and light scattering instruments. The typical elution profile of the two proteins, in this case the RI trace, is shown. The molecular weights of the protein across the elution profile is 28,670 and 26,800 Da for CS-peptide and G3 respectively. Given their similar molecule weights the relative elution positions of the two proteins indicate significant differences in solution structural behaviour.

Supplemental 3

```
MGSSHHHHHHSSGLVPRGSHMEFSGLPSGIAEVSGESSRAEIGSSLPSGAYYGSGTP
SSFPTVSLVDRTLVESVTQRPTAQEAGEGPSGILELSGAHSGAPDMSGEHSGFLDLS
GLQSGLIEPSGEPPGTPYFSGDFASTTNVSGESSVAMGTSGEASGLPEVTLITSEFV
EGVTEPTISQELGQRPPVTHTPQLFESSGKVSTAGDISGATPVLPGSGVEVSSVPES
SSETSAYPEAGFGASAAPEASREDSGSPDLSETTSAFHEANLERSSGLGVSGSTLTF
QEGEASAAPEVSGESTTTSDVGT
```

Amino Acid	% in sequence	Amino Acid	% in sequence
Serine	19.2	Isoleucine	2.5
Glycine	13.9	Glutamine	2.5
Glutamic Acid	11.0	Arginine	2.1
Threonine	8.9	Tyrosine	1.4
Proline	8.2	Histidine	1.1
Alanine	7.8	Asparagine	0.7
Valine	6.8	Methionine	0.7
Leucine	6.8	Lysine	0.3
Phenylalanine	3.2	Cysteine	0
Aspartic Acid	2.8	Tryptophan	0

S3: The amino acid sequence and relative amino acid composition of CS-peptide.

Supplemental 4

Glycosylation of CS2-G3

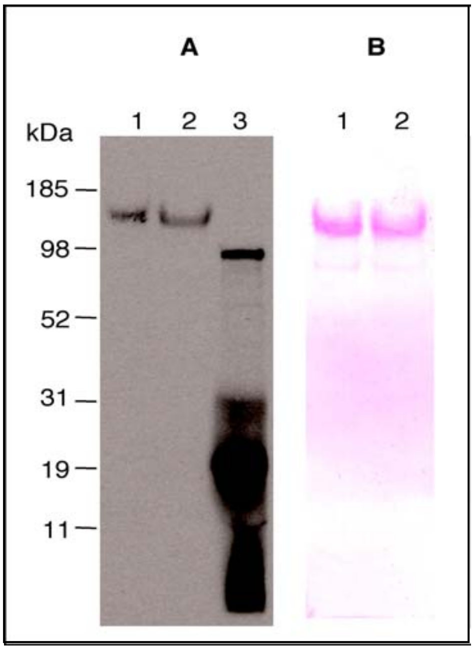

S4: A. Western blot with anti-G3 antibody of 1; untreated CS2-G3, 2; CS2-G3 treated with PNGaseF/EndoF, 3; TNT^{TM} cell-free expressed CS2-G3. B. PAS stain of 1; untreated CS2-G3, 2; CS2-G3 treated with PNGaseF/EndoF. The results indicate that there is glycosylation of the one N-linked site, and the cell-free expression, which has no capacity to glycosylate the expressed protein, indicates that there is approximately 25-30 kDa of O-linked sugar residues. The broad PAS stain at low molecular weight was non-specific and present in all lanes, including those not containing protein samples (not shown).

Methodology for cell-free expression

The CS2-G3 construct was taken from the *P. pastoris* vector pPICZαB (Invitrogen) and ligated into the pET-14b (Novagen) vector in-frame with the T7 promotor to create a plasmid for cell-free expression using TNT^{TM} (Promega). Expression of the construct was carried out according to the suppliers (supplier) instructions for a standard plasmid reaction, and incubated for 90 minutes before analysis of transcription/translation products by western blot.

Supplemental 5

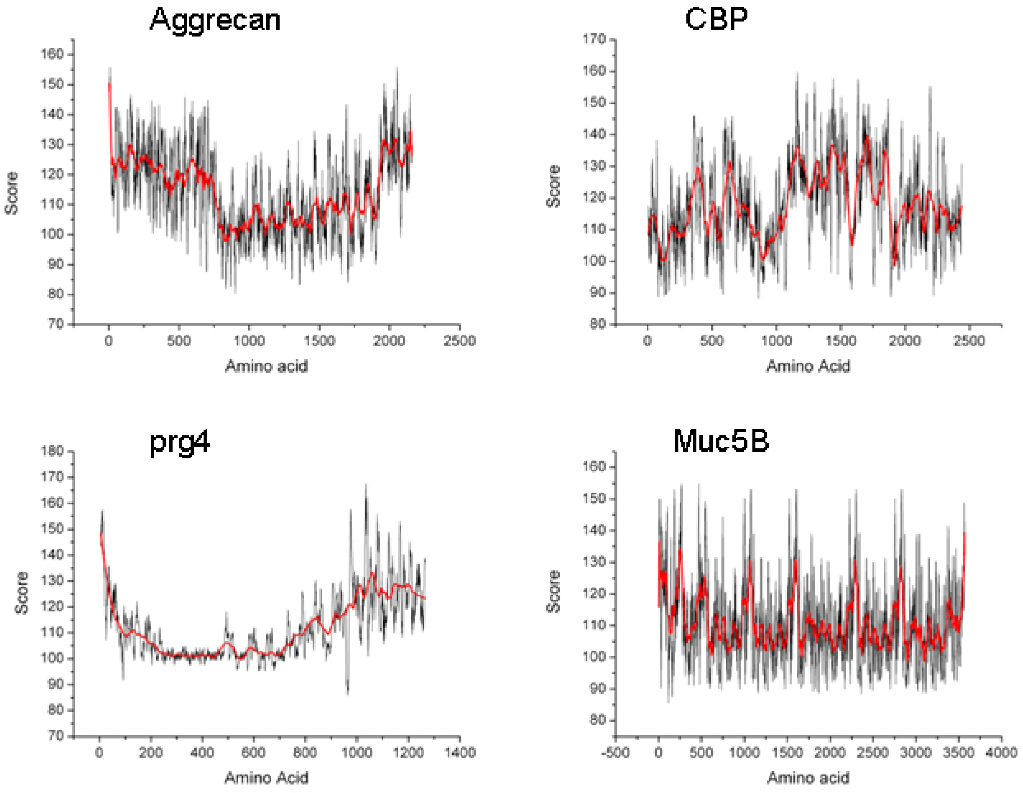

S5: Sequence analysis of aggrecan, CBP, prg4 and Muc5B proteins using the average area buried algorithm within the suite of programs in Protscale (http://www.expasy.ch/tools/protscale.html). The red lines are a nearest-neighbour average of 20 amino acids. The linker or extended sequences of these proteins can be clearly defined as having a low score (<115), whilst the more globular regions have a higher overall score (>115).
